# Growth performance, feeding behavior, serum biochemical, and meat quality traits of Karayaka lambs fed pastures consisting of different relative forage quality

**DOI:** 10.1007/s11250-026-04956-4

**Published:** 2026-03-02

**Authors:** Ahmet Akdağ, İbrahim Aydın, Nuh Ocak

**Affiliations:** 1 Faculty of Agriculture, Department of Animal Science, Eskisehir Osmangazi University, Eskişehir, Türkiye; 2 Faculty of Agriculture, Department of Field Crops, Ondokuz Mayis University, Samsun, Türkiye; 3 Faculty of Agriculture, Department of Animal Science, Ondokuz Mayis University, Samsun, Türkiye

**Keywords:** Forage quality, Grazing history, Growth rate, Lamb production, Metabolic profile, Nutritional value

## Abstract

This study evaluated the effects of pastures differing in relative forage quality (RFQ) on the growth, behavior, and meat quality of Karayaka lambs. Thirty-six male lambs (3-month-old and weighing 22.8 ± 0.14 kg) were assigned to four RFQ groups (*n* = 9) in a randomized complete block design. Lambs grazed for 60 days on pastures with RFQ indices of 89.3 (89RFQ), 105.2 (105RFQ), 121.4 (121RFQ), and 147.0 (147RFQ), each characterized by a distinct botanical composition. Growth and dry matter intake (DMI) were monitored every 10 days, while water intake was recorded daily. At the end of the trial, feeding behavior was assessed using a preference test, and serum biochemistry and meat quality were analyzed. The 121RFQ group showed a higher growth rate than the 105RFQ and 147RFQ groups (*p* < 0.05. DMI was highest at 121RFQ and lowest at 147RFQ (*p* < 0.05). The 121RFQ and 89RFQ lambs had better feed conversion ratios than 105RFQ (*p* < 0.05). The 105RFQ lambs consumed more water than the 121RFQ and 147RFQ lambs (*p* < 0.05). Grazing history influenced behavior: lambs preferred forages they had previously grazed (*p* < 0.05). The b* value of *longissimus dorsi* was higher in 121RFQ than in 147RFQ (*p* < 0.05). The 89RFQ and 105RFQ groups had higher meat fat and serum triglycerides, respectively (*p* < 0.05). In conclusion, although a higher RFQ was expected to maximize performance, 121RFQ yielded the best growth, indicating that botanical composition is a critical factor alongside quality index. Furthermore, grazing history significantly determines forage selection.

## Introduction

Pasture-based sheep production plays a significant economic and environmental role worldwide (Kemp et al. 2010; Poli et al. 2020; Costa et al. 2022). Furthermore, pasture-based lamb production reduces input costs, promotes ecological sustainability and animal welfare, and procures potentially healthier meat (Elizalde et al. 2021; Costa et al. 2022). However, lamb production remains far below its potential, primarily due to limited understanding of pasture quality and the nutrition of pasture-fed lambs, especially in tropical and subtropical regions (Papadopoulou et al. 2020; Poli et al. 2020). Additionally, wide variations in legume and grass content, driven by factors such as plant growth rate, seed ratio, establishment success, management, and persistence, make it challenging to control the quality and quantity of forage grazed by lambs (Poli et al. 2020; Costa et al. 2022). Therefore, these systems can result in slower lamb growth, delaying the attainment of the target weight and ultimately affecting production efficiency (Papadopoulou et al. 2020; Costa et al. 2022).

In pastoral systems, key challenges include improving animal performance, producing healthy, high-quality products, and maintaining the persistence and productivity of legumes and grasses. To address these challenges, there is growing recognition of the value of incorporating diverse forages into pasture establishment (Ates et al. 2013, 2015; Jiménez et al. 2019). Papadopoulos et al. (2001) showed that adding white clover (*Trifolium repens*) to orchardgrass (*Dactylis glomerata*) pastures improved lamb performance. Fraser et al. (2004) found that grazing lambs on forage-legume swards, such as red clover (*Trifolium pratense*) and alfalfa (*Medicago sativa*), enhances lamb performance and improves the unsaturated fatty acid composition of muscle without sacrificing carcass or meat quality, compared with grazing on perennial ryegrass (*Lolium perenne*) swards. Kemp et al. (2010) observed that pastures based on chicory (*Cichorium intybus*), plantain (*Plantago lanceolata*), red clover (*Trifolium pratense*), and white clover (*Trifolium repens*) resulted in higher body weight gain (BWG) in lambs than those based on perennial ryegrass (*Lolium perenne*). Incorporation of subterranean clover (*Trifolium subterraneum*) into pastures based on orchardgrass (*Dactylis glomerata*) and perennial ryegrass (*Lolium perenne*) has increased pasture production and lamb growth rates (Ates et al. 2013, 2015).

Based on the above research findings, animal productivity in grazing systems depends on forage species (such as legumes, grasses, and other plant groups) and on their characteristics (nutrient composition, quality, and quantity). Pasture forage quality, defined as the capacity of forage to supply the requisite nutrients for grazing animals, including lambs (Tesk et al. 2018), is a critical determinant of effective lamb production. Consequently, achieving optimal lamb performance is closely associated with specific nutrient quantities and key forage quality indicators (Tesk et al. 2018; Aydın et al. 2019), such as crude protein (CP), total digestible nutrients (TDN), digestible dry matter (DDM), and metabolizable energy (ME). One of the main constraints in grazing systems is limited dry matter intake (DMI) by grazing animals, which reduces their ability to meet nutrient requirements (including energy and protein) necessary for high performance (Fernandez-Turren et al. 2020). Relative forage quality (RFQ), used as a quality indicator (Aydin et al. 2019), is a crucial index for evaluating the nutritional value of ruminant feed (Favre et al. 2019). Since the RFQ index relies on neutral detergent fiber (NDF), NDF digestibility (NDFD), acid detergent fiber (ADF), TDN, CP, fatty acids, and ash (Favre et al. 2019), a pasture established according to this index can overcome this constraint on pasture quality.

To our knowledge, the effects of pastures composed of species with different RFQ indices on the growth performance, carcass traits, and meat quality of grazing lambs have not been investigated. Additionally, no information is available regarding the effects of such a pasture-based diet on specific blood parameters and meat quality in growing lambs. Negative experiences with novel forage species may reduce the acceptance of those species in subsequent encounters (Pedernera et al. 2022). However, when grazing animals are introduced to a variety of species, this exposure can reduce intensive grazing pressure on familiar, preferred forage species, thereby lowering the risk of pasture degradation caused by persistent overgrazing (Launchbaugh and Howery 2005). Moreover, grazing animals’ preference for unfamiliar and potentially low-quality forages may reduce their nutrient intake, thereby compromising their productivity and feeding behavior (Pedernera et al. 2022). However, the influence of prior experience, including grazing history, on the feeding preferences and behavior of lambs exposed to unfamiliar forages differing in RFQ indices has not yet been quantified. We hypothesized that lambs grazing on pastures established with higher RFQ would exhibit significantly improved growth performance, carcass traits, and meat quality, and that lambs with a prior history of diverse grazing would show greater adaptability and reduced aversion when exposed to novel forages from pastures with a different RFQ. Accordingly, the study aimed to (i) quantify production benefits for growing Karayaka male lambs grazed on pastures with different RFQ indices in terms of BWG, dry matter intake (DMI), feed conversion ratio (FCR), and carcass weight and yield, and (ii) investigate pH, color traits, and nutrient content of meat, as well as the effects of dietary history on plant preferences and feeding behavior of lambs exposed to unfamiliar pasture forages with different RFQ indices.

## Materials and methods

### Experimental site and design

This experiment was conducted from April 2019 to July 2020 in a 0.544-ha field at the Research and Application Farm, Bafra Station of the Ondokuz Mayıs University (40° 59’ 40” N, 35° 54’ 27” E, 8 m a.s.l.). The primary soil characteristics of the experimental site were loamy texture, pH 7.73; workability 55%; organic matter 0.60%; total salt 0.009% (as NaCl); lime 10.9% (CaCO_3_); P 0.66 kg/da (as P_2_O_5_); and K 25 kg/da (as K_2_O). The experimental site’s climate is typical of the Black Sea coast, characterized by year-round humidity and rainfall, hot summers, and mild winters. During the experiment, the monthly averages of temperature, precipitation, and humidity were 14.6 °C, 716.7 mm, and 73.1%, respectively. In contrast, during the grazing period, the corresponding values were 21.0 °C, 15.0 mm, and 69.5%.

In the first year of this two-year study, on April 15, 2019, the experimental area was fenced. A systemic herbicide, Roundup Star (Bayer Türk Kimya San. Ltd. Şti., Istanbul, Turkiye), containing 441 g/L glyphosate (potassium salt) in a water-soluble formulation, was applied immediately at a rate of 3 L/ha, using a backpack sprayer to control existing perennial and hard-to-control weeds. Soil preparation on May 5, 2019, included tillage (15–20 cm) to achieve a fine, well-levelled seedbed and the incorporation of fertilizers (20 kg/ha of each N, P, and K) based on soil analysis results.

**Fig. 1:**
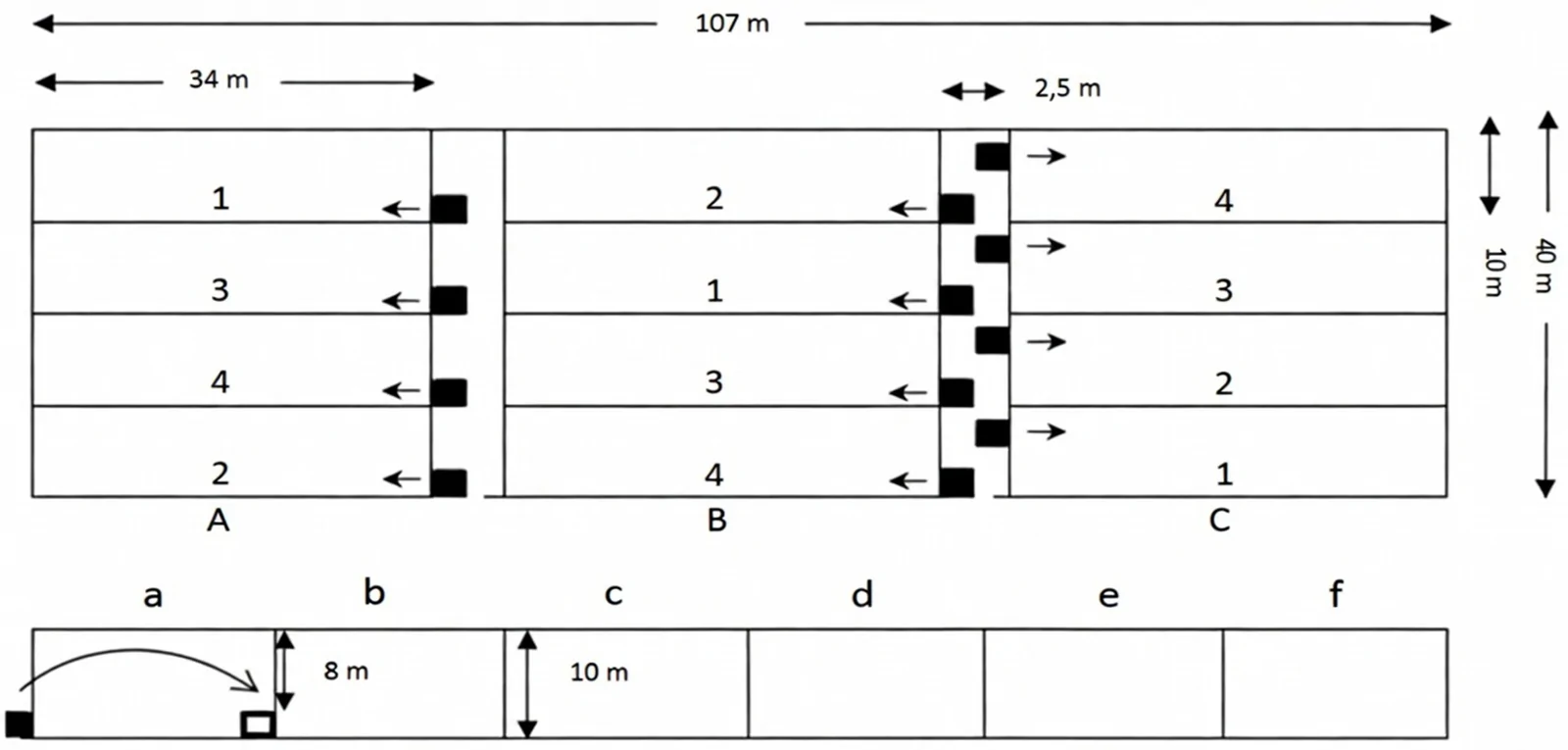
The blocks and subplots of pastures established under different relative forage quality and grazing management practices. A, B and C represent the main plots; 1, 2, 3 and 4 indicate the relative feed quality (RFQ) treatments; ■ denotes the shelters; a, b, c, d, e and f represent the subplots served as replicates and a 10-day grazing pattern was implemented in each subplot

The experiment was conducted in a randomized complete block design. Therefore, the experimental area was fenced to separate the three original blocks (replicates), each measuring 0.136 ha. Each block was subsequently subdivided into four subplots (each 0.034 ha). The size of each subplot was determined to meet the dry matter (DM) requirements of three lambs during a 60-day grazing period. This calculation was based on the nutritional requirements for lambs (NRC 2007), assuming a dry matter intake (DMI) of 3.5% of body weight (BW). The four RFQ treatments, detailed below, were randomly assigned to these subplots (Fig. 1).

### Species selection and pasture establishment

To establish pastures to achieve the target RFQ indices (89.3, 105.2, 121.4, and 147.0), plant species were selected based on high forage yield and persistence, as determined in a previous study conducted at the same station (Aydın et al. 2022). Specific single-species or species mixtures were formulated to meet each RFQ target based on the RFQ of the individual components (Table 1). The RFQ index for each established pasture (treatment) was calculated as follows:

**Table 1:** Plant species and their relative forage quality (RFQ) that were used for establishing the experimental pastures, and the inclusion rate (%) of each species in the formation of RFQ treatments

Species	RFQ^1^	RFQ of the established pasture (treatment)^2^			
		89.3 (89RFQ)	105.2 (105RFQ)	121.4 (121RFQ)	147.0 (147RFQ)
Bird’s foot trefoil (*Lotus corniculatus* Population)	147				100 (147.0)
Alfalfa (*Medicago sativa cv*. Emiliana)	129		40 (51.6)		
White clover (*Trifolium repens* cv. Rivandel)	126			80 (100.8)	
Perennial ryegrass (*Lolium perenne* cv. Belida)	103			20 (20.6)	
Meadow fescue (*Festuca pratensis* Population)	92	33.3 (30.6)^3^	20 (18.4)		
Tall fescue (*Festuca arundinacea* cv. Starlett)	91	33.3 (30.4)	20 (18.2)		
Cocksfoot grass (*Dactylis glomerata* cv. Lidacta)	85	33.3 (28.3)	20 (17.0)		

^1^The RFQ indices for individual species were determined by Aydın et al. (2022)

^2^The pasture RFQ indices that were calculated as the sum of the products of the RFQ index of each species and its corresponding inclusion rate in the seed mixture, and the names (codes in parentheses) of the established pastures (RFQ treatment)

^3^Values in parentheses represent the RFQ amount supplied by each species in the seed mixture

$$\:{RFQ}_{Pasture}=\sum\limits_{i=1}^{n}\left({RFQ}_{i}\right)\:\times\:{P}_{i})$$

Wherein: RFQ_pasture_ denotes the final RFQ for the established pasture. RFQi represents the RFQ of the ith species in the mixture. Pi denotes the inclusion rate of the ith species in the seed mixture (g per 100 g). n represents the total number of species in the seed mixture. The comprehensive chemical analyses (including NDF, NDFD, TDN, fatty acids, and ash) required to calculate the RFQ indices for individual species growing at the same station were previously conducted by the same research group. The results were reported by Aydın et al. (2022). To ensure methodological consistency, these previously determined RFQ indices were adopted for the current study.

On May 6, 2019, sowing was performed simultaneously across all subplots using a drill to ensure precise placement of the adapted forage mixture at a depth of 2–3 cm. For each RFQ treatment, the seeding rate per hectare for each component species was calculated from base sowing rates of 20 kg/ha for legumes and 30 kg/ha for grasses, to provide the required rate of pure, viable seed in each mixture. For the second application, approximately three weeks after seedling emergence, equal amounts of fertilizer (20 kg/ha each of N, P, and K) were manually applied to all subplots. Irrigation was applied twice—once during the tillering stage and once during the stem elongation stage—and as needed during later stages. If weeding was performed, it was carried out continuously and uniformly by hand across all subplots throughout the experiment.

### Lambs and grazing management

Thirty-six intact male Karayaka lambs, three months old and weighing 22.8 ± 0.14 kg, were transferred to the experimental area from the Sheep Production Unit of the Research and Application Farm, Faculty of Agriculture, Ondokuz Mayıs University. The experiment lasted for 74 days, comprising 14 days of adaptation to the experimental condition and 60 days of data collection. During the adaptation period, internal and external parasite control were implemented by orally administering half a tablet of the anthelmintic Dicromec^®^ (Damla İlaç ve Kimya San. Tic. Ltd. Şti., Istanbul, Turkiye) to each lamb (equivalent to 0.2 mg ivermectin/kg BW and 5 mg praziquantel/kg BW). Doramectin (Dectomax^®^, Zoetis Deutschland GmbH, Germany) was administered subcutaneously at a dose of 0.46 mL per lamb. To determine the initial BW (on day 1) of the experiment, the lambs were weighed individually on May 2, 2020, using a sensitive electronic scale with 100 g precision (Immax EB-600 ovine livestock scale, Istanbul, Turkiye). The lambs were then randomly allocated to one of 12 subplots (three lambs per subplot), each of which was assigned to one of the four RFQ pastures. The subplots, separated by temporary fencing, were rotationally grazed every 10 days for 60 days (Fig. 1). Self-measuring drinkers (plastic-calibrated, 10-liter Smartware buckets; Turan Plastik Smartware, Istanbul, Turkiye) and commercial mineral blocks (Mineral Blok; Royal İlaç^®^, Kayseri, Turkiye) were suspended in the shelter cabins to provide ad libitum access to potable water and meet the lambs’ mineral requirements, respectively. All calibrated buckets, including blank buckets, were filled daily with freshwater, and the daily reduction in water volume was measured. A three-sided wooden shelter (1.5 m × 2 m × 2 m) serving as the lambs’ overnight resting area was relocated to the next grazing subplot under rotational grazing management.

### Growth performance and feeding behavior

To monitor BW, BWG, and, particularly, DM requirements, all lambs were weighed on days 10, 20, 30, 40, 50, and 60 during the experiment. Before weighing, lambs were fasted for 14 h to minimize the effect of gastrointestinal tract fill on their BW. The BWG for each lamb was calculated for three distinct periods. The average daily BWG for the first and last 30-day periods was determined by subtracting the BW on day 1 from the BW on day 30 and the BW on day 31 from the BW on day 60, respectively, then dividing each difference by 30. The weight gain over 60 days was calculated by subtracting the BW on day 1 from the BW on day 60, then dividing the difference by 60. The DMI for the lambs in each subplot was estimated from pre- and post-grazing herbage mass measurements taken at 10-day intervals using the cage technique (Fraser et al. 2004; Undi et al. 2008). Because lambs in each subplot were grazed as a group, BW, BWG, and DMI data are presented as mean values for the three lambs per subplot and used to calculate the feed conversion ratio (FCR). Thus, the FCR was calculated as the ratio of average DMI to average BWG for each 30-day phase and the entire 60-day experimental period. Daily water intake (DWI) was recorded by comparing the water offered to the amount remaining in the calibrated plastic buckets, and accounting for potential water loss due to evaporation as measured in an identical blank bucket (actual DWI = apparent DWI of the lambs minus evaporation loss).

Feeding behavior was observed and recorded from 07:30 to 09:30 h on three consecutive days, as described by Rice et al. (2016). A free-choice trial was conducted to determine the influence of grazing history on lambs’ forage preference. One lamb per subplot (*n* = 3 per RFQ treatment) was exposed to unfamiliar forages from different RFQ pastures during the three days following the experimental period. Forages (each with an RFQ index of 500 g) were offered simultaneously in four troughs to the selected lambs, which were housed in individual pens. The primary outcome measures recorded were first-choice feed selection and total feeding time. Camera recordings were used to determine the total time lambs from each plot spent in optional feeders containing forages with different RFQ values, and the time spent seeking feed before the first definite intake. Finally, the total time spent at each feeder and the first-choice selections were expressed as percentages of the relevant total number of observations.

At the end of the 60-day grazing period, all lambs were removed from their RFQ treatments, fasted for 12 h, and weighed again to determine slaughter weight (SW) before transport to slaughter. From each subplot, two lambs with BW closest to the treatment mean (*n* = 6 per treatment) were slaughtered according to Halal-certified procedures at a commercial abattoir (Bafra Commodity Exchange Slaughterhouse Limited, Samsun, Turkiye). This procedure involves a single swift cut, severing the carotid arteries, jugular veins, trachea, and esophagus; it is performed without prior stunning to ensure rapid blood loss. After slaughter, rumen pH was measured, without opening the rumen, using a HI 8521 glass electrode pH meter (Hanna Instruments Deutschland GmbH, Karlsruhe, Germany) to determine whether it was affected by forages with different RFQ indices. Immediately after we removed the internal organs, we measured carcass weights. Carcass yield (dressing percentage) was calculated as the ratio of carcass weight to SW.

### Blood biochemistry

To assess serum biochemical parameters, 10 mL of blood was collected from the jugular vein of selected lambs (*n* = 6 per treatment) at slaughter and transferred into Vacutainer^®^ tubes containing ethylenediaminetetraacetic acid (EDTA). The samples were collected between 08:00 and 09:00 h to minimize diurnal and feeding-related effects on blood metabolites following an overnight fast. The samples were then centrifuged at 3,000 × g for 15 min at 4 °C. The serum was separated into clean, dry 2-mL Eppendorf tubes and frozen at − 20 °C until analysis. Serum samples were analyzed on a Biosystem autoanalyzer (Roche Integra^®^ 400 plus, Roche Diagnostics GmbH, Mannheim, Germany) to determine the concentrations of glucose, blood urea nitrogen (BUN), triglycerides, Ca, and P, using specific kits and following the manufacturer’s instructions. Additionally, enzyme activities, such as alkaline phosphatase (ALP), alanine aminotransferase (ALT), and aspartate aminotransferase (AST), were determined using a Biosystems A25 autoanalyzer (Biosystems S.A., Barcelona, Spain) by the UV-kinetic method (International Federation of Clinical Chemistry, Milan, Italy).

### Meat quality

*Longissimus dorsi* muscle (LDM) samples from two carcasses per replicate were used to assess meat quality parameters, including pH, color, and nutrient content. The pH of the LDM at the interface between the 12th and 13th ribs was measured at 1 h (pH_1_) and 24 h (pH_24_) after slaughter using a Testo 205 pH meter (Testo AG, Lenzkirch, Germany) with a solid glass probe. Color traits in the LDM were assessed at these time points using the CIE L* (lightness), a* (redness), and b* (yellowness) system with a Minolta CR-400 colorimeter (Minolta Camera Co., Osaka, Japan). Three replicate measurements were taken at each time point. The meat samples kept at 4 °C for 24 h were subjected to analytical procedures. A change in the weight of the LDM over the subsequent 24 h was taken as the drip loss (%), as described by Bond and Warner (2007). The contents of DM (method 930.15), ash (method 942.15), CP (method 990.03), and ether extract (method 920.39) in the LDM were determined in triplicate using the approved methods (AOAC 2005).

### Pasture characterization

To analyze the chemical composition (NDF, ADF, CP, IVDMD) and calculate the quality indicators (TDN and ME) of the RFQ pastures (Table 2), herbage samples were hand-harvested from each subplot at 10-day intervals. Samples were air-dried to constant weight at room temperature and ground. The same analytical procedures and equipment, including an ANKOM A200/220 Fiber Analyser (ANKOM Technology Corp., Fairport, NY, USA) and an ANKOM Daisy II in vitro incubator (ANKOM Technology, Macedon, NY, USA), were employed as described by Aydın et al. (2019).

**Table 2:** Chemical composition and some quality indicators of pastures with different relative forage quality (RFQ) during the first (days 1 to 30) and the last (days 31 to 60) 30 days of the grazing period

Item^1^	Days 1 to 30					Days 31 to 60				
	89RFQ	105RFQ	121RFQ	147RFQ		89RFQ	105RFQ	121RFQ	147RFQ	
DM (%)	29.78	24.30	26.29	22.09		32.40	28.45	22.45	25.02	
Nutrient composition (% of DM)										
CP	12.14	20.30	17.09	19.49		12.66	18.91	19.27	19.59	
ADF	32.24	29.25	30.66	28.37		36.37	33.23	29.80	32.11	
NDF	58.09	48.91	49.71	44.16		66.49	50.02	47.20	48.91	
Ash	10.62	11.79	9.94	10.58		11.89	11.25	10.58	11.79	
Quality indicator (% of DM)										
IVDMD	45.36	49.15	48.78	53.00		39.07	48.56	51.14	53.11	
TDN	58.93	65.36	64.49	68.04		58.26	62.45	66.24	64.70	
ME, Mj/kg DM	8.91	9.52	9.08	8.30		8.59	9.16	8.90	8.36	

89RFQ: 33.3% meadow fescue, 33.3% tall fescue, 33.3% cocksfoot grass; 105RFQ: 40% alfalfa, 20% meadow fescue, 20% tall fescue, 20% cocksfoot grass; 121RFQ: 80% white clover, 20% perennial ryegrass; 147RFQ: 100% bird’s-foot trefoil. DM: Dry matter, CP: Crude protein, ADF: Acid detergent fiber, NDF: Neutral detergent fiber, IVDMD: In vitro dry matter digestibility, TDN: Total digestible nutrients, ME: Metabolizable energy

^1^ The values represent the means of the analyses and calculations performed with the samples obtained at 10-day intervals during the 60-day grazing period

### Statistics

All data were analyzed by calculating mean values of measurements within each subplot and by applying the MIXED procedure in SPSS v. 21.0 (SPSS Inc., Chicago, IL, USA). Before analysis, normality and homogeneity of the data were assessed using the Kolmogorov–Smirnov and Levene tests, respectively. No outliers were detected. Percentage data that were not normally distributed were arcsine-transformed. Data were initially intended for evaluation using a two-way repeated-measures analysis of variance. Although the data met the assumption of normality, Mauchly’s test of sphericity indicated a violation of the sphericity assumption (*p* < 0.05). Consequently, to maintain a robust statistical approach and account for the nested structure of the subplots, the data were analyzed using the following model:

$$\:{{\rm\:Y}}_{ij\:}=\:\mu\:+\:{\alpha\:}_{i\:}+\:{{\upbeta\:}}_{i\left(j\right)\:}+\:{\epsilon}_{ij\:}$$

Wherein: Υij = the value referring to the observation of the jth treatment within the ith block; µ = the overall mean; αi​ = the effect of the ith block (i = A, B, C); βj(i) = the effect of the jth RFQ treatment within the ith block (j = 1, 2, 3, 4); ϵij = the random error. The means were compared using Tukey’s HSD test at a significance level of *p* ≤ 0.05.

## Results

### Growth performance

The BW of the lambs differed significantly among RFQ pastures on days 30 (*p* = 0.045) and 60 (*p* = 0.047) of the experiment (Table 3). The 89RFQ lambs on day 30 and 121RFQ lambs on day 60 had higher BW than the 105RFQ and 147RFQ lambs (*p* < 0.05). During the first 30 days, daily BWG was higher in the 89RFQ pasture than in the 105RFQ and 147RFQ pastures (*p* < 0.05). The 121RFQ lambs consumed more DM than the 105RFQ and 147RFQ lambs (*p* < 0.05). The 89RFQ lambs exhibited a lower FCR than other pasture lambs (*p* < 0.05). The DWI of the 105RFQ lambs was higher than that of the 147RFQ lambs. During the second 30-day phase (days 31–60), 21RFQ lambs exhibited a higher daily BWG than 105RFQ lambs (*p* < 0.05). Daily DMI in the 147RFQ pasture was lower than in the 89RFQ, 105RFQ, and 121RFQ pastures (*p* < 0.05). The RCR in the 21RFQ and 147RFQ pastures was lower than that in the 105RFQ pasture (*p* < 0.05). During this period, the DWI of the 105RFQ lambs was higher than that of the 147RFQ lambs. Over the entire 60-day grazing period, the daily BWG of the 121RFQ lambs exceeded those of the 105RFQ and 147RFQ lambs (*p* < 0.05). The 121RFQ lambs had the highest daily DMI; the 89RFQ and 105RFQ lambs had intermediate daily DMI, while the 147RFQ lambs had the lowest daily DMI (*p* < 0.05). The 121RFQ and 89RFQ lambs exhibited a better FCR than the 105RFQ pasture (*p* < 0.05). The DWI of the 105RFQ lambs was higher than that of the 121RFQ and 147RFQ lambs (*p* < 0.05).

**Table 3:** Growth performance of Karayaka male lambs grazing on pastures with different relative forage quality (RFQ)

Variable^1^	89RFQ	105RFQ	121RFQ	147RFQ	SEM	*p*-value
Body weight (kg) on day 1	22.93	22.90	22.66	22.86	0.136	0.928
Body weight (kg) on day 30	27.43^a^	24.30^b^	24.56^ab^	24.14^b^	0.237	0.045
Body weight (kg) on day 60	27.61^ab^	26.18^b^	28.28^a^	26.53^b^	0.390	0.047
1 to 30 days						
Body weight gain (g/day)	73.78^a^	47.13^b^	63.47^ab^	42.34^b^	9.834	0.043
Dry matter intake (g/day)	705.5^ab^	655.8^b^	861.5^a^	593.5^b^	37.26	0.027
Feed conversion ratio (g/g)	9.56^b^	13.91^a^	13.57^a^	14.01^a^	0.695	0.029
Water intake (L/day)	0.87^ab^	1.17^a^	0.75^ab^	0.64^b^	0.083	0.039
31 to 60 days						
Body weight gain (g/day)	82.22^ab^	62.20^b^	123.86^a^	79.99^ab^	9.007	0.036
Dry matter intake (g/day)	963.0^a^	975.7^a^	990.7^a^	845.8^b^	20.65	0.017
Feed conversion ratio (g/g)	11.71^ab^	15.68^a^	7.99^b^	10.56^b^	0.871	0.036
Water intake (L/day)	1.31^ab^	1.43^a^	1.19^ab^	1.04^b^	0.043	0.037
1 to 60 days						
Body weight gain (g/day)	78.05^ab^	54.55^b^	95.83^a^	63.33^b^	6.397	0.031
Dry matter intake (g/day)	834.2^b^	815.7^b^	926.1^a^	719.7^c^	24.84	0.004
Feed conversion ratio (g/g)	11.16^b^	14.89^a^	9.88^b^	11.74^ab^	0.725	0.040
Water intake (L/day)	1.09^ab^	1.30^a^	0.97^b^	0.84^b^	0.062	0.029

89RFQ: 33.3% meadow fescue, 33.3% tall fescue, 33.3% cocksfoot grass; 105RFQ: 40% alfalfa, 20% meadow fescue, 20% tall fescue, 20% cocksfoot grass; 121RFQ: 80% white clover, 20% perennial ryegrass; 147RFQ: 100% bird’s-foot trefoil. SEM: Standard error of the mean

^a,b,c^ Mean values in the same row with different superscripts differ (*p <* 0.05)

^1^ Values are means of three subplots with three lambs per treatment

### Carcass and meat quality traits

No significant differences were observed among RFQ treatments for SW, carcass weight, or carcass yield; however, rumen pH differed significantly (Table 4). The rumen pH of the 147RFQ lambs was lower than that of the 89RFQ, 105RFQ, and 12RFQ lambs (*p* < 0.05). The RFQ treatments did not affect the studied meat quality traits (*p* > 0.05), except for the b* values at 1 h (*p* = 0.023) and 24 h (*p* = 0.040) and the fat content of the LDM at 24 h post-slaughter (*p* = 0.031; Table 5). While its b* values at 1 h post-slaughter were higher in the 89RFQ lambs than in the 105RFQ and 147RFQ lambs (*p* < 0.05), the corresponding b* values at 24 h post-slaughter were higher in the 121RFQ lambs than in the 147RFQ lambs (*p* < 0.05). The 89RFQ and 105RFQ pastures resulted in higher muscle fat content at 24 h post-slaughter than the 121RFQ and 147RFQ pastures (*p* < 0.05).

**Table 4:** The weight of slaughter (SW) and carcass, carcass yield and rumen pH value of Karayaka male lambs grazing on pastures with different relative forage quality (RFQ)

Variable^1^	89RFQ	105RFQ	121RFQ	147RFQ	SEM	*p*-value
Slaughter weight (kg)	27.61	26.28	28.41	26.46	0.390	0.167
Carcass weight (kg)	10.70	10.32	11.18	10.37	0.164	0.236
Carcass yield (% of SW)	38.76	39.30	39.36	39.23	0.001	0.517
Rumen pH	6.37^ab^	6.09^ab^	6.55^a^	5.70^c^	0.109	0.004

89RFQ: 33.3% meadow fescue, 33.3% tall fescue, 33.3% cocksfoot grass; 105RFQ: 40% alfalfa, 20% meadow fescue, 20% tall fescue, 20% cocksfoot grass; 121RFQ: 80% white clover, 20% perennial ryegrass; 147RFQ: 100% bird’s-foot trefoil. SEM: Standard error of the mean

^a,b,c^ Mean values in the row with different superscripts differ (*p <* 0.05)

^1^ Values are means of three subplots with two lambs per treatment

**Table 5:** Meat quality traits of the *longissimus dorsi* muscle of lambs grazing on pastures with different relative forage quality (RFQ)

Variable^1^	89RFQ	105RFQ	121RFQ	147RFQ	SEM	*p*-value
One hour after slaughter						
pH_1_	5.94	6.11	5.86	6.12	0.067	0.488
CIELab values						
Lightness (L*)	38.42	38.76	38.09	38.42	0.549	0.982
Redness (a*)	15.28	14.36	13.72	13.93	0.278	0.202
Yellowness (b*)	5.42^a^	4.04^b^	4.50^ab^	3.86^b^	0.203	0.023
24 h after slaughter						
pH_24_	5.64	5.66	5.58	5.65	0.023	0.659
CIELab values						
Lightness (L*)	42.29	43.77	44.65	44.68	0.546	0.379
Redness (a*)	14.58	14.91	13.62	14.03	0.233	0.213
Yellowness (b*)	7.57^ab^	7.75^ab^	9.38^a^	7.83^b^	0.288	0.040
Drip loss, %	0.62	0.54	0.80	0.47	0.113	0.813
Chemical composition, % of dry matter						
Dry matter, %	24.42	24.53	23.36	22.76	0.392	0.346
Ash	1.53	1.61	1.71	1.69	0.044	0.539
Protein	20.41	20.51	19.90	19.98	0.206	0.717
Fat	2.47^a^	2.40^a^	1.08^b^	1.73^b^	0.086	0.031

89RFQ: 33.3% meadow fescue, 33.3% tall fescue, 33.3% cocksfoot grass; 105RFQ: 40% alfalfa, 20% meadow fescue, 20% tall fescue, 20% cocksfoot grass; 121RFQ: 80% white clover, 20% perennial ryegrass; 147RFQ: 100% bird’s-foot trefoil. SEM: Standard error of the mean

^a,b,c^ Mean values in the same row with different superscripts differ (*p <* 0.05)

^1^ Values are means of three subplots with two lambs per treatment

### Blood biochemistry

The RQF treatments did not significantly affect serum glucose and Ca concentrations or the activities of the studied enzymes (Table 6). The 105RFQ lambs had higher serum triglyceride levels than the 147RFQ lambs (*p* < 0.05). The 89RFQ pasture resulted in lower BUN concentration but higher serum P content compared with the 105RFQ and 121RFQ pastures (*p* < 0.05).

**Table 6:** Serum biochemical parameters of Karayaka male lambs grazing on pastures with different relative forage quality (RFQ)

Variable^1^	89RFQ	105RFQ	121RFQ	147RFQ	SEM	*p*-value
Serum metabolic profile (mg/dL)						
Glucose	64.66	60.00	66.33	71.00	1.892	0.239
Triglycerides	27.66^ab^	38.00^a^	23.66^ab^	19.33^b^	2.976	0.012
Blood urea nitrogen	11.33^b^	20.66^a^	19.66^a^	17.66^ab^	1.257	0.008
Calcium	9.60	9.53	9.76	9.20	0.141	0.612
Phosphorus	5.80^a^	4.33^b^	4.53^b^	4.76^ab^	0.232	0.018
Enzyme activity (U/L)						
Alkaline phosphatase	124.00	106.00	126.00	136.00	16.313	0.234
Alanine aminotransferase	10.00	8.33	10.66	11.33	0.743	0.585
Aspartate aminotransaminase	75.00	80.00	84.33	75.00	3.412	0.833

89RFQ: 33.3% meadow fescue, 33.3% tall fescue, 33.3% cocksfoot grass; 105RFQ: 40% alfalfa, 20% meadow fescue, 20% tall fescue, 20% cocksfoot grass; 121RFQ: 80% white clover, 20% perennial ryegrass; 147RFQ: 100% bird’s-foot trefoil. SEM: Standard error of the mean

^a,b,c^ Mean values in the same row with different superscripts differ (*p <* 0.05)

^1^ Values are means of three subplots with two lambs per treatment

### Feeding behavior

The lambs previously grazed on 89RFQ pastures spent a similar percentage of time in the 89RFQ troughs as in the 105RFQ troughs (Table 7). They spent more time in both of these in accordance with the 121RFQ and 147RFQ troughs (*p* < 0.05). Lambs with a history of 105RFQ grazing spent the most time at 105RFQ troughs and a similar amount of time at 121RFQ troughs; they spent significantly more time at both than at 89RFQ and 147RFQ troughs (*p* < 0.05). Lambs from the 121RFQ group spent the most time in the 105RFQ troughs, followed by time in the 121RFQ, 89RFQ, and 147RFQ troughs (*p* < 0.05). A similar pattern was observed for the 147RFQ lambs, which spent the most time in the 105RFQ troughs, followed by the 147RFQ, 121RFQ, and 89RFQ troughs (*p* < 0.05). The lambs’ initial feeding preferences were clearly influenced by their prior dietary experience (Table 7). Lambs previously fed 89RFQ forage overwhelmingly preferred 105RFQ forage to the familiar 89RFQ forage. Similarly, lambs previously fed 105RFQ showed a strong preference for 89RFQ forage over 147RFQ forage. Lambs that had previously consumed 121RFQ demonstrated a strong preference for this familiar forage. Finally, lambs with prior exposure to 147RFQ preferred the familiar 147RFQ forage over the unfamiliar 105RFQ forage.

**Table 7:** The influence of grazing history on forage preference by lambs exposed to unfamiliar forages and the percentage of first-choice forages from each relative forage quality (RFQ) pasture

Lamb	Grazing history					
	89RFQ	105RFQ	121RFQ	147RFQ	SEM	*p*-value
Time spent in the optional troughs (%)^2^						
89RFQ	26.27^ab^ (22.2)	27.59^a^ (77.8)	24.81^b^	21.52^c^	0.617	0.024
105RFQ	21.74^bc^	34.41^a^	25.00^b^ (44.4)	18.84^c^ (55.6)	1.192	0.006
121RFQ	5.92^d^	43.18^a^	33.64^b^ (100)	17.27^c^	2.692	< 0.001
147RFQ	8.01^c^	45.24^a^	23.71^b^ (22.2)	22.61^b^ (77.8)	2.504	< 0.001

89RFQ: 33.3% meadow fescue, 33.3% tall fescue, 33.3% cocksfoot grass; 105RFQ: 40% alfalfa, 20% meadow fescue, 20% tall fescue, 20% cocksfoot grass; 121RFQ: 80% white clover, 20% perennial ryegrass; 147RFQ: 100% bird’s-foot trefoil. SEM: Standard error of the mean

^a,b,c^ Mean values in the same row with different superscripts differ (*p <* 0.05)

^1^ Values are the average of three replicates with two lambs per treatment selected at the end of the growth trial

^2^ Percentage of the total time spent by the lambs in the troughs. Values ​​in parentheses indicate the percentage of first-choice forages from the RFQ pastures

## Discussion

The findings of the present study indicate that the RFQ index, a robust tool for categorizing forage quality based on chemical composition and fiber digestibility (Aydin et al. 2019; Favre et al. 2019), may not reliably predict lamb growth performance in grazing systems. The lack of a linear relationship between RFQ indices and lamb performance in forage-fed ruminants may reflect complex interactions among factors, including botanical composition, palatability, nutrient synchrony, and post-ingestive feedback (Chen et al. 2022; Pepeta et al. 2022; Tahir et al. 2022). These factors are not captured by the parameters (CP, ADF, NDF, TDN, and NDFD) integrated into the RFQ equation (Favre et al. 2019). This composition likely increased total fiber content and induced physical satiety, thereby limiting DMI and reducing overall nutrient utilization efficiency in the lambs. Therefore, the predictive accuracy of RFQ for lamb production can be enhanced by incorporating additional variables, such as forage palatability, nutrient availability, synchrony, and complex interactions within pasture vegetation (Pepeta et al. 2022; Tahir et al. 2022), rather than relying solely on chemical indices.

As this is the first study to evaluate these parameters in lambs grazed on pastures with different RFQ indices, our findings are interpreted in light of research on lambs in extensive grazing systems or on lambs receiving forage-based diets. Our growth performance results align with established literature, indicating that while forage mass significantly influences intake (Turner et al. 2014), botanical composition, particularly the inclusion of legumes, has a greater effect on dietary protein utilization and growth rates because legumes have faster digestion kinetics (Kaithwas et al. 2020; Tahir et al. 2022). Furthermore, comparable growth across different forage species, as observed in previous studies (Fraser et al. 2004; Ates et al. 2013, 2015), has been attributed to similarities in forage quality, supporting the notion that specific forage characteristics beyond a broad index such as RFQ are the primary determinants of animal performance.

Uzun and Ocak (2019) noted that pastured animal productivity is contingent on grazing management (frequency, intensity, and pressure) and forage characteristics (botanical composition, quality, and quantity). Poor lamb performance may primarily result from nutritional deficiencies caused by feeding roughage as the sole dietary source (Elizalde et al. 2021). Variations in forage quality are mainly associated with fiber and CP content, while the limiting factor for DMI is NDF intake (Fernandez-Turren et al. 2020). This result may explain why Karayaka lambs performed poorly in our study compared with previous studies using the same breed (Olfaz et al. 2005; Sen et al. 2011; Yıldırım et al. 2013). Indeed, when animals grazed legume-based pastures, they tended to exhibit higher BWG and carcass weights than animals that grazed grass-based pastures (Fraser et al. 2004; Kemp et al. 2010). Therefore, future models that predict lamb performance in pasture-based systems should integrate detailed forage compositional data alongside quality indices to enhance accuracy and practical applicability.

The superior growth performance of the 121RFQ lambs suggests that the blend of white clover and perennial ryegrass provided a palatable and nutritionally balanced diet conducive to growth, despite its nutrient values not being uniformly highest across all nutritional parameters. Bird’s-foot trefoil typically has a higher cell-to-wall ratio and accumulates greater concentrations of soluble sugars; these physiological characteristics contribute to a superior RFQ index compared with those of grass species (Aydin et al. 2019). Accordingly, the pronounced decline in ruminal pH observed in the highest RFQ treatment likely resulted from the rapid fermentation of high levels of water-soluble carbohydrates, leading to elevated volatile fatty acid concentrations (Kolver and de Veth 2002). Although comprehensive ruminal metabolomic profiling was not performed in this study, the observed shift toward a more acidic environment may have exceeded the rumen’s physiological buffering capacity. According to Kolver and de Veth (2002), a ruminal pH of 5.8–6.2 is sufficient to maintain performance in pasture-based systems. This benchmark clarifies why the 121RFQ treatment, which maintained a more balanced ruminal environment, sustained superior growth performance compared with the 147RFQ treatment, where the pH dropped below this critical threshold, potentially impairing microbial synthesis. This acidification likely compromised the activity of cellulolytic microbiota, thereby impairing digestive efficiency and inducing metabolic feedback that depressed DMI and subsequent growth rates (Ren et al. 2023), even for forages characterized by superior quality indices.

Papadopoulos et al. (2001) and Kemp et al. (2010) reported that mixtures of white clover and wheat had effects on lamb growth performance similar to those observed in the present study. The disparity in the FCR across groups indicates that efficient nutrient utilization is not solely determined by estimated energy content (Birkett de Lange, 2001). In particular, the presence of specific botanical components may have optimized metabolic efficiency independently of the calculated RFQ value. Moreover, ryegrass-white clover mixtures (up to 50%) outperformed ryegrass monocultures (Niderkorn et al. 2017). This supports the idea that polyculture pastures are advantageous for DMI and the preservation of forage quality (Ates et al. 2015; Jiménez et al. 2019). This observation may be related to increased nutrient utilization and reduced environmental impacts through associative interactions among plant chemical constituents (Mueller-Harvey et al. 2019). Furthermore, a high proportion of grass species was associated with low growth rate and high FCR, as reported by Kaithwas et al. (2020) and Tahir et al. (2022).

The discrepancy between high CP levels in pure stands of bird’s-foot trefoil and the subsequent depression of lambs’ DMI suggests that intrinsic forage factors, such as palatability or post-ingestive feedback, may override standard quality indices. This intake limitation is probably due to the unique fermentation profile (Bach et al. 2005) of a monoculture of bird’s-foot trefoil, which can differ greatly from that of multi-species RFQ treatments. While standard indices like RFQ classify such forages as high-quality (Aydın et al. 2019), they may not capture the metabolic changes induced by grazing a single species. Furthermore, the lack of improvement in growth performance observed in this study challenges previous assertions that tannin-containing legumes (Girard et al. 2016) necessarily enhance BWG through superior protein bypass and utilization compared to traditional legume-grass mixtures. These findings highlight a complex interaction in which specific plant secondary metabolites and fermentation kinetics can counteract the expected nutritional advantages of high RFQ, underscoring the importance of integrated evaluation models that go beyond chemical analysis alone.

Contrary to expectations that improved forage quality, usually marked by higher protein and lower fiber content, would affect physiological water demand (Malan et al. 2020), our results instead indicate that DWI is influenced by a different factor. The higher DWI recorded in certain groups appears more closely associated with the immediate water status of the pasture vegetation, specifically its DM content and canopy surface moisture, than with its inherent nutritional density (Sun et al. 2014). This indicates that environmental and physical attributes of the forage may exert a more immediate influence on lambs’ DWI than chemical quality indices alone. This outcome suggests that pasture-related environmental factors may exert a more immediate influence on lambs’ water consumption than the forage’s inherent nutritional quality (Malan et al. 2020). Consequently, the present results on DWI have potentially significant implications for our understanding of the complex interplay among forage RFQ levels, DMI, and DWI in grazing lambs. Further investigation is warranted to elucidate osmotic and metabolic mechanisms by which specific forage compositional attributes may ultimately influence water intake patterns.

The nutritional results for forages across the grazing period demonstrated temporal dynamics in key nutrients, particularly CP. The shift in CP content from the initial to the later phase underscores the importance of considering forage maturity and botanical composition rather than relying on a static RFQ value. The nutritive value and nutrient degradation kinetics of pastures are influenced by botanical composition, harvest date, and the interaction between pasture type and harvest date (Keim et al. 2013; Ma et al. 2021). Although a consistent inverse relationship (Roukos et al. 2011; Aydin et al. 2019) exists between RFQ and fiber fractions (NDF and ADF), the forage mixes with higher RFQ indices may have contained components that hindered IVDMD (Moore and Jung 2001). Because of the non-linear relationship between RFQ and ME (Aydın et al. 2019), estimating energy availability for growing lambs based on this index alone is difficult.

Varying forage compositions elicited distinct metabolic responses, as evidenced by fluctuations in mineral metabolite levels. For instance, systemic phosphorus accumulation appears to result directly from high mineral intake from grass-based pastures rather than reflecting the overall RFQ index (Mueller-Harvey et al. 2019). These findings suggest that varying forage compositions elicited distinct metabolic responses, highlighting RFQ’s limitations in predicting specific nutrient absorption and utilization at a biochemical level. The observed positive correlation between CP and BUN, consistent with previous reports (Li et al. 2025), indicates that increased nitrogen intake is associated with higher BUN levels. However, elevated BUN, as suggested by Kohn et al. (2005), may also indicate reduced nitrogen utilization efficiency. These selective biochemical responses underscore the need to consider forage composition beyond RFQ to fully understand nutrient dynamics in grazing lambs.

The selective impact of RFQ on meat quality, specifically on b* values and intramuscular fat, suggests subtle forage-induced alterations in muscle metabolism. Although RFQ treatments significantly affected fat content, they did not alter meat color, despite an inverse correlation between fat content and b* values, with lower fat levels associated with higher b* values (Calnan et al. 2017). This suggests that the consistently observed pH values, rather than variations in fat content, are more likely to account for the lack of significant differences in meat color (Montelli et al. 2021). The tendency of lower-RFQ forages to promote intramuscular fat deposition may be linked to a higher ruminal acetate-to-propionate ratio. Indeed, Ren et al. (2023) reported that, in sheep fed only forage, increases in total volatile fatty acids and in the ruminal acetate-to-propionic-acid ratio resulted in greater intramuscular fat content, despite lower overall performance. This metabolic partitioning suggests that forages with higher fiber fractions can effectively support marbling, even when overall growth performance is not maximized (Lin et al. 2020; Ren et al. 2023). However, further research is needed to confirm this biochemical mechanism and its impact on fatty acid profiles and quality. The muscle nutrient content is consistent with previous studies on Karayaka lambs (Olfaz et al. 2005; Aksoy et al. 2019). However, the elevated intramuscular fat content (1.08%–2.47%) in grazing Karayaka lambs, regardless of RFQ treatment, may reflect a genetic predisposition to intramuscular fat deposition (marbling) resulting from distinctive metabolic partitioning of energy (Aksoy et al. 2019). This finding, therefore, highlights a specific quality attribute of this native breed (Olfaz et al. 2005; Sen et al. 2011; Yıldırım et al. 2013).

Lambs’ forage preferences are learned, with familiarity and positive post-ingestive feedback significantly influencing feed selection (Pedernera et al. 2022). Lambs preferred forages with RFQ values similar to those in their grazing history, suggesting that familiarity and gradual changes in forage quality play a role. Consistent with Fitzsimons et al. (2017), feeding behavior is driven by forage quantity and quality and mediated by physical and metabolic mechanisms shaped by novel forage characteristics. While trough-seeking time remained consistent (4.35 ± 0.426) across RFQ groups, the time spent at troughs varied with RFQ and indicated that selective feeding decisions were based on prior experience and forage characteristics (Baumont et al. 2000; Neave et al. 2018). This highlights that both immediate availability and experience influence feeding behavior. Dietary diversity or complementary forage combinations may better support lamb growth than RFQ alone (Pedernera et al. 2022). However, initial palatability can sometimes override established familiarity. The findings confirm that prior experience enhances foraging efficiency (Launchbaugh and Howery 2005; Neave et al. 2018) and that variation in time spent at different RFQ troughs may reflect nutritional rebalancing (Baumont et al. 2000). The introduction of grazing ruminants into novel environments containing unfamiliar or undesirable plant species can precipitate a decline in pasture quality and health, driven by the selective overconsumption of habitual forage (Launchbaugh and Howery 2005; Neave et al. 2018; Pedernera et al. 2022). It is imperative to investigate further and recognize the implications of these observations for the sustainability and resilience of pastoral systems.

### Limitations

Despite the insights gained, this study has several limitations. A significant constraint is the relatively small sample size per treatment (*n* = 9 lambs; 3 per subplot), which may have reduced statistical power and increased sensitivity to distributional variation. Moreover, the absence of a direct digestibility trial and the measurement of only rumen pH, without assessing other metabolites such as volatile fatty acids or NH3-N, limits our understanding of the dynamic physiological processes influencing traits such as intramuscular fat content. Notably, while the RFQ index served as a valuable tool for categorizing forage based on chemical parameters, it did not fully account for the decisive role of botanical composition in determining animal outcomes. The lack of integration of factors such as species-specific digestion kinetics, palatability, and nutrient synchrony into the RFQ equation represents a conceptual limitation in predicting performance within complex grazing systems. Consequently, these results should be interpreted with caution, and the observed trends require confirmation in future studies with larger cohorts, longer durations, and more comprehensive metabolic profiling across varied climatic conditions.

## Conclusion

This study evaluated the effects of varying RFQ indices on growth, feeding behavior, and meat quality in Karayaka lambs to identify an optimal quality threshold. The results demonstrate that the 121RFQ pasture provides the most effective balance between maximizing BWG and feed conversion efficiency. Although an RFQ value of 147 was expected to yield superior results, the specific botanical composition and ruminal environment, particularly the maintenance of pH within the optimal physiological range, proved more decisive than the RFQ index alone. Our results confirm that a balanced legume-grass mixture provides a more stable nutritional environment for lamb growth than monoculture stands. Therefore, pasture management should prioritise optimized mixture compositions that ensure high nutritional quality and animal acceptability. Furthermore, grazing history was confirmed as a significant determinant of forage preferences, influencing lambs’ adaptation to varying pasture quality. Managing pastures to achieve an RFQ target of approximately 121, while considering botanical diversity, is recommended to optimize growth performance in Karayaka lambs. Future research should focus on the long-term effects of these quality thresholds on ruminal microbial ecology and nutrient digestibility.

## Data Availability

The data for this study are available from the corresponding author upon reasonable request.
